# Application of adipose tissue-derived stem cell therapy with a clinically relevant dose does not significantly affect atherosclerotic plaque characteristics in a streptozotocin-induced hyperglycaemia mouse model

**DOI:** 10.1016/j.jmccpl.2024.100083

**Published:** 2024-07-09

**Authors:** Amber Korn, Suat Simsek, Mitchell D. Fiet, Ingeborg S.E. Waas, Hans W.M. Niessen, Paul A.J. Krijnen

**Affiliations:** a Department of Pathology, Amsterdam University Medical Centres (AUMC), Location VUmc, Amsterdam, the Netherlands; b Department of Internal Medicine, Northwest Clinics, Alkmaar, the Netherlands; c Department of Internal Medicine, AUMC, Location VUmc, Amsterdam, the Netherlands; d Amsterdam Cardiovascular Sciences, Amsterdam, the Netherlands; e Department of Pathology, AUMC, Location AMC, Amsterdam, the Netherlands; f Department of Cardiac Surgery, AUMC, Location VUmc, Amsterdam, the Netherlands

**Keywords:** Adipose tissue-derived stem cells, ApoE^−/−^ mouse, Atherosclerosis, Circulating monocytes, Diabetes mellitus, Immunohisochemistry, Mesenchymal stem cell therapy

## Abstract

**Aims:**

Diabetes mellitus (DM) induces increased inflammation of atherosclerotic plaques, resulting in elevated plaque instability. Mesenchymal stem cell (MSC) therapy was shown to decrease plaque size and increase stability in non-DM animal models. We now studied the effect of MSC therapy in a streptozotocin-induced hyperglycaemia mouse model using a clinically relevant dose of adipose tissue-derived MSCs (ASCs).

**Methods:**

Hyperglycaemia was induced in male C57BL/6 ApoE^−/−^ mice (*n*=24) via intraperitoneal streptozotocin (STZ) injection (0.05 mg/g bodyweight) for 5 consecutive days. 16 weeks after the first STZ injection, the mice received either 100,000 ASCs (*n*=9) or vehicle (*n*=14) intravenously. The effects of ASC treatment on the size and stability of aortic root atherosclerotic plaques were determined 4 weeks post-treatment via (immuno)histochemical analyses. Furthermore, plasma monocyte subsets within 3 days pre- and 3 days post-treatment, and 4 weeks post-treatment, were studied.

**Results:**

ASC treatment did not significantly affect atherosclerotic plaque size or intra-plaque inflammation. Although ASC-treated mice had a higher percentage of intra-plaque fibrosis (42.5±3.3%) compared to vehicle-treated mice (37.6±6.8%, *p*=0.07), this did not reach significance. Additionally, although differences in the percentages of circulating pro- and anti-inflammatory monocytes were observed after ASC treatment compared to pre-treatment (*p*=0.005), their levels did not differ significantly at any time point compared to vehicle-treated mice.

**Conclusions:**

ASC treatment with a clinically relevant dose did not significantly affect atherosclerotic plaque size or intra-plaque inflammation in a hyperglycaemia mouse model. Despite a borderline significant improvement in intraplaque fibrotic content, the potential of ASC treatment on atherosclerotic plaque stability in a diabetic environment remains to be determined.

## Graphical abstract

**Figure:**
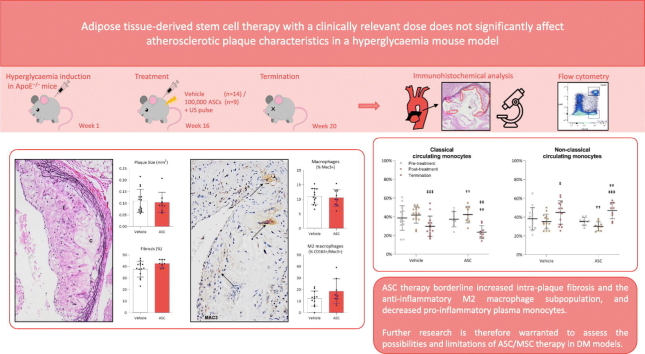

## 1 Introduction

Diabetes mellitus (DM) is a substantial global issue, that was affecting approximately 463 million people in 2019 and is only expected to rise further [1]. DM, both type I and type II, are characterized by poor glycaemic control which results in hyperglycaemia. Within the diabetic population, the main cause of morbidity and mortality is cardiovascular disease wherein atherosclerosis plays a major role [[2], [3], [4]]. Atherosclerotic plaque formation is a consequence of chronic cholesterol accumulation in the vascular wall, mainly within arterial vessels. Recruited immune cells and inflammation play a crucial part both in plaque development and plaque complications. Intra-plaque inflammation is considered to be a major driver of atherosclerotic plaque complication, in part by thinning the fibrous cap, making the plaques more prone to rupture [5]. It has been shown that in atherosclerotic plaques of patients with DM inflammation and calcification is increased [6]. In addition, patients with DM and acute coronary syndrome had a higher prevalence of lipid-rich plaques with high macrophage accumulation, a thinner fibrotic cap and increased plaque instability throughout the coronary network compared to the non-DM control patients [7]. Therapeutic targeting of these vascular complications may attenuate the development of cardiovascular disease.

Mesenchymal stem cells (MSCs) have been shown to be an effective therapy in a variety of cardiovascular diseases [8,9]. These adult multipotent stem cells can be harvested from multiple sites, including bone marrow and adipose tissue, and can modulate the immune system in several ways, including polarising macrophages towards the anti-inflammatory M2 phenotype and producing anti-inflammatory cytokines [10,11]. Although bone marrow derived MSCs (BMSCs) have been used for MSC therapy traditionally, they have an invasive harvesting procedure with a relative low yield. Adipose tissue-derived MSCs (ASCs) are thus increasingly investigated, as they can be obtained more easily with a higher yield while still demonstrating the immunomodulating properties [11,12]. These properties make MSCs theoretically suitable to combat atherosclerosis. In pre-clinical studies BMSC therapy attenuated the development and size of atherosclerotic plaques, as well as plaque instability, in non-DM animal models [9,13]. In diabetic mice, systemic BMSC application decreased hyperglycaemia and nephropathy [14]. However, while DM-associated complications (i.e., nephro-, neuro- and retinopathy) are increasingly investigated, there are no studies yet describing the effect of MSC therapy on atherosclerotic plaques of the aorta in a diabetic environment.

It is generally recognized that MSC therapy has been less effective in patients than in mice [15,16]. While there may be several explanations for this, one reason could be the disproportionally high numbers of MSC that are applied in murine studies compared to human studies. In murine models usually around 1–1.5 million BMSCs per mouse are applied intravenously [13,14], whereas in clinical trials in which BMSCs or ASCs are given intravenously, generally around 1–4 million MSCs/kg of bodyweight are applied [[17], [18], [19], [20], [21]]. Assuming a mouse weighs approximately 25 grams, an MSC dose proportionate to clinically applied doses would then be in the range of 25,000–100,000 MSCs per mouse.

We thus investigated how ASC therapy affects atherosclerotic plaques in a hyperglycaemic mouse model using a clinically relevant dose of ASCs. We hypothesized that ASC therapy improves atherosclerotic plaque characteristics due to its immunomodulatory effects, possibly with a lower effect due to the clinically relevant dose.

## 2 Materials and methods

### 2.1 Stem cell isolation

The stromal vascular fraction (SVF) was harvested from adipose tissue isolated from the abdominal subcutis and inguinal fat pads of 5–7 weeks old healthy male C57BL/6 mice (*n*=5; Charles River, The Netherlands) and stored in liquid nitrogen as described previously [22]. To obtain the adipose tissue-derived stem cells (ASCs) the SVF was thawed and seeded in selective MesenCult Basal Medium (Stemcell Technologies) supplemented with 10% MesenCult Supplement and 0.1% MesenPure (Stemcell Technologies) one week before injection. The ASCs were cultured at 37°C and medium was refreshed every 2–3 days. On the day of treatment, the cultured ASCs were harvested using trypsin/EDTA and resuspended in serum-free Dulbecco's Modified Eagle Medium (DMEM) with a final concentration of 100,000 cells/100 μL.

### 2.2 Animal procedures

**Fig. 1:**
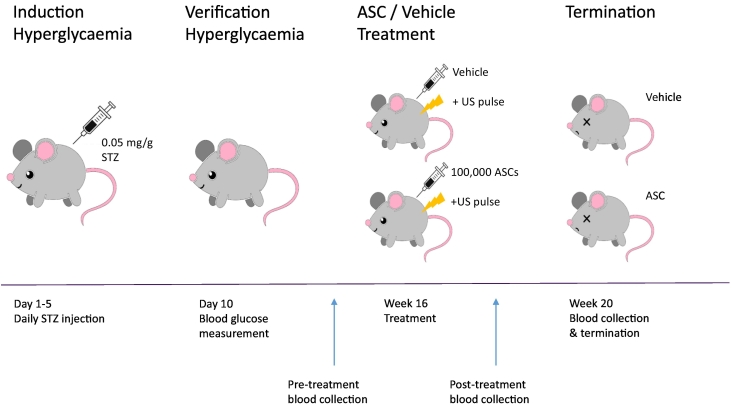
Schematic overview of animal experiment. Male C57/Bl6 ApoE^−/−^ mice were injected with 0.05 mg/g bodyweight STZ on five consecutive days to induce hyperglycaemia, which was verified on day 10 using blood glucose measurements. 16 weeks post-STZ injection the mice received a single intravenous injection with or without 100,000 stem cells under 2% isoflurane anaesthesia, followed by a 1min ultrasound pulse, resulting in a stem cell-treated group (*n*=9) and a vehicle-treated group (*n*=14) respectively. Blood samples were obtained from the tail vein within 3 days pre- and post-treatment, and immediately prior to termination 4 weeks after treatment via cardiac punction.

Hyperglycaemia was induced in 24 6–8 weeks old healthy male C57BL/6 ApoE^−/−^ mice [23,24] by injecting 0.05 mg/g bodyweight streptozotocin (STZ) intraperitoneally for 5 consecutive days (Fig. 1). Blood glucose levels were measured 10 days after the first STZ injection (Table 1). Successful hyperglycaemic induction was defined as a blood glucose level of >10 mmol/L, and 96% of the mice developed DM (23/24 mice). Throughout the experiment the mice were fed normal chow ad libitum. Body weight was measured 2–3 times weekly until termination. 16 weeks after the first STZ injection, the mice were anesthetized with 2% isoflurane and either 100,000 ASCs (*n*=9) or vehicle (*n*=14) were infused slowly into the tail vein. As this experiment was part of a larger animal experiment in which ultrasound pulsation was employed analysing the so called StemBells [25], ASC treatment was immediately followed by a 1min pulse of 1 MHz ultrasound with an acoustic pressure of 100 kPa and a 1 kHz pulse repetition frequency, positioning the transducer (V303-SU, Panametrics) parasternal directed at the anterior wall of the heart. The transducer was coupled to a waveform generator (33220A, Agilent) and a linear 60-dB power amplifier (150A100B, Amplifier Research). Blood samples were obtained from the tail vein within 3 days before and after ASC treatment, and immediately prior to termination 4 weeks after ASC treatment via cardiac punction. After termination, the heart and the connected thoracic aorta were excised for immunohistochemical analysis.

**Table 1:** Bodyweight and blood glucose levels of all mice included. Bodyweight presented in grams and blood glucose levels presented in mmol/L of mice treated with either vehicle or stem cell therapy. Measurements were made prior to the first streptozotocin injection (start) and at termination (end), blood glucose levels were additionally measured within 3 days before and after treatment (pre- and post-treatment respectively). Experimental groups were compared on the same day (not significant), and between days (displayed as* for difference compared to start value). Data is displayed as mean±SD. *P*<0.05*, p<0.01**, p<0.001***.

Treatment	Body weight (g)		Blood glucose (mmol/L)			
	Start	End	Start	Pre-treatment	Post-treatment	End
Vehicle	25.3±1.1	24.9±3.9	21.2±4.3	27.1±2.6***	26.8±3.5***	26.9±3.2***
ASCs	23.9±1.6	25.7±3.3	21.8±4.6	26.3±4.4***	26.3±4.4***	26.2±4.8***

This study was approved by the Central Committee for Animal Experiments of the Netherlands (the Centrale Commissie Dierproeven) and performed according to the guidelines of the animal ethics committee of the VU University, Amsterdam.

### 2.3 Tissue processing

Tissue was immediately fixated in formalin and subsequently embedded in paraffin. The top of the heart, containing the aortic root, was embedded separately from the rest of the heart. The paraffin-embedded heart top was then serially cut into 4 μm-thick sections. To specify the location of the aortic root (beginning of the aorta containing the aortic valves), every 20th section was mounted onto a microscope slide and stained with a haematoxylin-eosin (HE) staining. The aortic root is approximately 0.24 mm long, so a range of 78 sections was used for further analysis to ensure the 60 sections (240 μm) containing the aortic root were fully included. Every 13th section of the 78-section range was mounted onto glass slide serially (section 1 on slide 1, section 2 on slide 2, and so on, then section 13 on slide 1, etc.), resulting in 13 glass slides with 6 sections each. These slides were then used for (immuno)histochemical analysis of plaque size, plaque stability and intra-plaque inflammation.

### 2.4 (Immuno)histochemical staining

First, the slides were deparaffinised, rehydrated and incubated with 0.3% H_2_O_2_ in methanol to block endogenous peroxidases. Heat-inactivating antigen retrieval was performed by boiling tissue in citrate buffer (pH 6.0; for ICAM-1, CD45, CD163 and Mac3 staining), or by incubating with 0.25% pepsin solution in 0.1mol/L HCl at 37°C (for Ly6G staining) for 10min. Antibodies were diluted in Tris-HCL-buffered saline (TBS) containing 1% BSA and 0.1% Tween (TBT) (for ICAM-1), or in normal antibody diluent (NAD) (for CD45, CD163, Ly6G, Mac3), and the incubations were performed at room temperature unless otherwise specified. For CD45, Ly6G and Mac3 staining, the slides were pre-incubated with normal rabbit serum (1:50, Dako) for 10min. For CD163 staining the slides were blocked with normal mouse serum (1:10, Jackson Immunoresearch) for 10min, whilst the ICAM-1 staining did not require blocking. Subsequently, the slides were incubated with rabbit-anti-mouse ICAM-1 (1:500, Abcam), rat-anti-mouse CD45 (1:50, BD Biosciences), rat-anti-mouse Ly6G (1:200, BD Biosciences) for 1h or with rat-anti-mouse Mac3 (1:30, Bioscience) at 4°C overnight. For the CD163 staining, a 1:200 antibody complex was constructed by incubating mouse-anti-mouse CD163 (1:200; Dako), rabbit-anti-mouse IgG antibody (Rockland), and NAD for 20min. Then normal mouse serum was added or 10min, and slides were incubated for 1h for CD163 staining. The slides were then incubated with the secondary antibodies rabbit-anti-rat biotin (1:400, Dako; for ICAM-1 staining), mouse-anti-rat biotin (1:100, Jackson ImmunoResearch; for CD45 and Ly6G staining) or with rabbit-anti-rat HRP (1:50, Dako; for CD163 and Mac3 staining) for 30min. For ICAM-1 staining, the slides were then incubated with ABC-kit (1:100, Vectorstain) for 30min, and for CD45 and Ly6G staining the slides were incubated with streptavidin HRP (1:100, Dako) for 60min. Following visualization with 3,3′ diaminobenzidine (DAB; Dako) for 1–3min for ICAM-1 staining and 7–10min for CD45 (leukocytes), CD163 (anti-inflammatory M2 macrophages), Mac3 (pan-macrophages) and Ly6G staining (neutrophilic granulocytes), the slides were counterstained with haematoxylin, dehydrated and covered.

For Toluidine staining (mast cells), the slides were first deparaffinised and rehydrated, then incubated with Toluidine Blue (1:100, gift from Molecular Diagnostics Department, AUMC, Location AMC). After incubation with 70% ethanol for 15s, the slides were dehydrated, and covered. The histochemical staining with Haematoxylin-Eosin (HE) and Elastica von Giesson (EvG) were performed according to standard protocol [25].

### 2.5 (Immuno)histochemical scoring

For the scoring of the markers, the slides were scanned using a Phillips slide scanner. Plaque size (mm^2^) was determined by encircling the atherosclerotic plaques using Philips IntelliSite Pathology Solution v3.2 software, only including tissue sections wherein the aortic valves were opened until sections wherein the valves were no longer visible. The mean plaque area was calculated per mouse. The percentage of aortic root endothelium positive for ICAM-1 was determined by quantifying ICAM-1 staining within the encircled atherosclerotic plaque endothelium. For the quantification of the intra-plaque cells, the atherosclerotic plaques were encircled in QuPath v.0.1.2 software and transferred to ImageJ v1.51g software where the areas stained positive for the inflammatory cell markers, as well as the total plaque areas, were measured. Only the mast cells (Toluidine Blue) were counted manually. The areas positive for the inflammatory cell markers were calculated as a percentage of the atherosclerotic plaque area or, in case of the number of mast cells, as the number of cells/mm^2^ within the atherosclerotic plaque.

### 2.6 PBMC isolation

Immediately after blood collection, the pre- and post-treatment blood samples (diluted 1/1 PBS) and the heart punction samples (undiluted) were transferred to Ficoll solution (Lymphoprep) and centrifuged at 1000×*g* at 4°C without brake for 20min. The peripheral blood mononuclear cell (PBMC) -containing layer was carefully aspirated, diluted with PBS and centrifuged at 250×*g* at 4°C for 10min. The supernatant was discarded and the PBMCs were resuspended in 0.5–1 mL Fetal Bovine Serum (FBS) containing 10% DMSO. PBMCs were immediately frozen in the −80°C freezer and stored in liquid nitrogen the following day.

### 2.7 Flow cytometry

The percentages of classical and non-classical monocytes in the blood were determined using flow cytometry. For this the thawed PBMCs were resuspended in 250 μL FACS buffer (0.5% BSA, 0.02% NaN3, and 2 mmol/L EDTA in PBS) containing 2.5 μL FITC-conjugated anti-CD11b antibody (eBioscience) and 2.5 μL APC-conjugated anti-Ly6C antibody (eBioscience) and incubated on ice for 30min. 1 mL FACS buffer was added to the PBMCs, followed by centrifugation at 600×*g* at 4°C for 8min. The supernatant was discarded and the PBMCs were resuspended in 200 μL FACS buffer and then analysed via flow cytometry (BD FACSCanto™ II with FloJo v10.7.1 analysis software). Among the CD11b+ monocytes two major subpopulations were identified according to their expression level of Ly6C: Ly6C^hi^ (classical monocytes) and Ly6C^low^ (non-classical monocytes).

### 2.8 Statistical analysis

Data analysis was performed with Prism v.4.0 (Graphpad Software, La Jolla, CA). Gaussian distribution was assessed with a Shapiro-Wilk normality test. Differences between groups were analysed with an unpaired *t*-test if the data was normally distributed and with a Mann-Whitney test in case of non-normally distributed data. Differences within groups were analysed with a paired t-test or a Wilcoxon matched-pairs signed rank test, if the data was respectively normally or non-normally distributed. Data values are displayed as mean±standard deviation and *p*<0.05 values (two-sided) were considered significant for all analyses.

## 3 Results

### 3.1 Body weight and blood glucose measurements

There were no significant differences in body weight between the vehicle-treated and ASC-treated groups at the start and end of the experiment, nor between start weight and end weight within the groups (Table 1).

At day 10 post-STZ, the blood glucose levels were 21.2±4.3 mmol/L in the vehicle-treated control group and 21.8±4.6 mmol/L in the ASC-treated group. In both the vehicle-treated and ASC-treated groups the blood glucose levels were significantly higher at pre- (*p*=0.0005 and *p*=0.008 respectively) and post-treatment time points (*p*=0.001 and p=0.008 respectively), as well as at termination (26.9±3.2 mmol/L, p=0.001; and 26.2±4.8 mmol/L, p=0.008 respectively) compared to day 10 post-STZ. No differences in glucose levels were found between vehicle- and ASC-treated animals at the different time points.

### 3.2 Atherosclerotic plaque size, stability and inflammation analyses

**Fig. 2:**
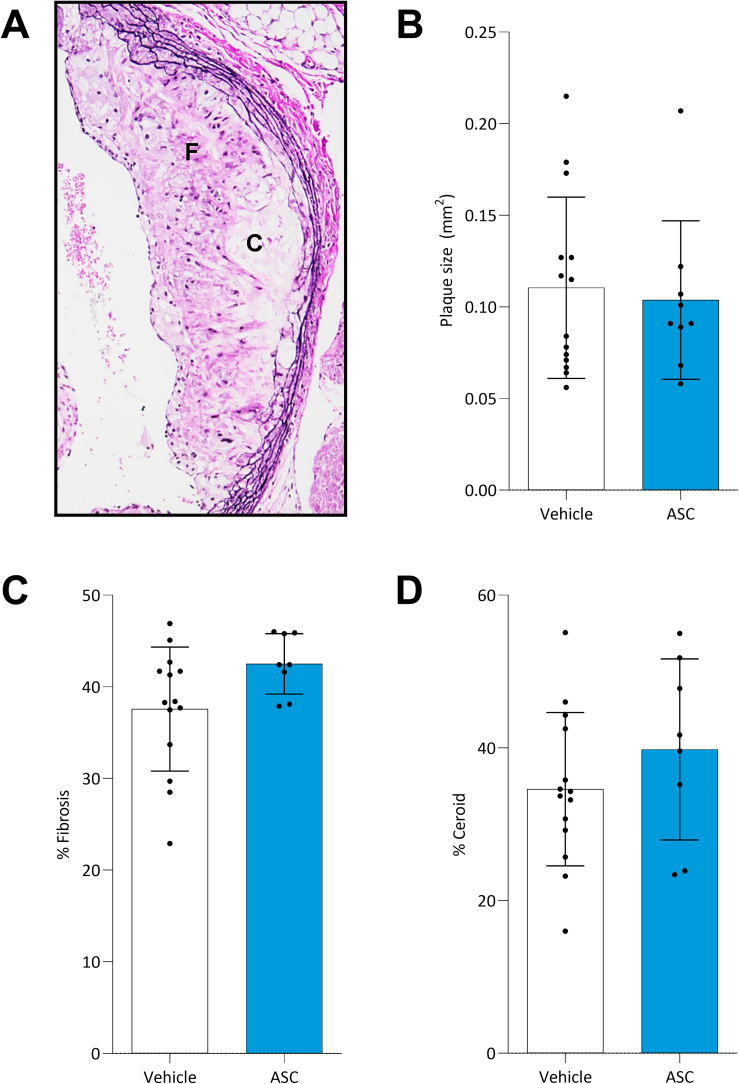
ASC therapy had little effect on atherosclerotic plaque stability in hyperglycaemic mice. Atherosclerotic plaque characteristics were examined in 14 vehicle-treated and 9 ASC-treated mice. Atherosclerotic plaques were included for histochemical analysis from the moment the aortic valves opened to the moment the aortic valves were no longer visible. Example of EvG staining of the atherosclerotic plaque in the aortic root (A; original magnification x100). The mean plaque size (mm^2^) was analysed based on the EvG staining (B), as well as the percentage of intra-plaque fibrosis (C) and ceroid (D), indicated by F and C respectively. An unpaired *t*-test was used for analysis, data is represented as mean±SD. Significance was reached with a *p*-value<0.05*.

All mice developed atherosclerotic plaques in the aortic root (Fig. 2A). No significant difference in plaque size was observed between the vehicle-treated (0.11±0.04 mm^2^) and the ASC-treated mice (0.10±0.05 mm^2^, *p*=0.9) (Fig. 2B). However, ASC therapy seemed to positively affect plaque stability as the percentage of intra-plaque fibrosis was higher in the stem-cell treated mice (42.5±3.3%, *p*=0.07) compared to the vehicle-treated mice (37.6±6.8%), albeit without reaching statistical significance (Fig. 2C). The percentage of intra-plaque ceroid in the ASC-treated mice (39.8±11.9%) and the vehicle-treated mice (34.6±10.0%, *p*=0.3) did not differ significantly (Fig. 2D).

**Fig. 3:**
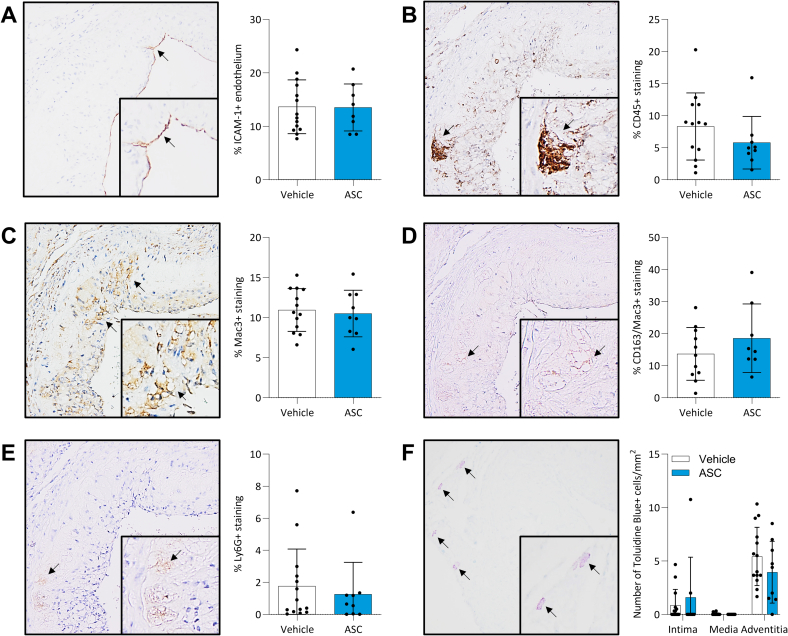
ASC therapy had minor effects on atherosclerotic plaque inflammation in hyperglycaemic mice. The inflammatory markers were assessed through immunohistochemical staining (A-F; original magnification x200 and x400), only including atherosclerotic plaques from the moment the aortic valves opened to the moment the aortic valves were no longer visible. In 14 vehicle-treated and 9 ASC-treated mice, the percentage of ICAM-1+ endothelium (A), and intra-plaque leukocytes (CD45; B), total macrophages (Mac3; C) and neutrophilic granulocytes (Ly6G; E) was quantified. Also the percentage of intra-plaque M2 macrophages (CD163) was quantified and presented as percentage of Mac3 staining (D). Additionally, the number of Toluidine Blue+ mast cells was determined in the intima, media and adventitia of the aortic root (F). An unpaired t-test was used for analysis, data is represented as mean±SD. Significance was reached with a p-value<0.05*. (For interpretation of the references to colour in this figure legend, the reader is referred to the web version of this article.)

We next analysed plaque inflammation. The percentages of ICAM-1+ endothelium (Fig. 3A**)**, CD45+ leukocytes (Fig. 3B), Mac3+ macrophages (Fig. 3C), CD163+ subpopulation macrophages (Fig. 3D), Ly6G+ neutrophils (Fig. 3E) and mast cells (Fig. 3F) did not differ significantly between the vehicle- and ASC-treated mice. Although the average percentages of intimal CD45+ leukocytes (5.8±4.1%; Fig. 3B) and adventitial mast cells (4.0±2.9%; Fig. 3F) in the ASC group were lower than in the vehicle group (CD45: 8.3±5.2%; mast cells: 5.4±2.7%), this did not reach statistical significance (*p*=0.2).

### 3.3 Percentages of circulating classical and non-classical monocyte subsets

**Fig. 4:**
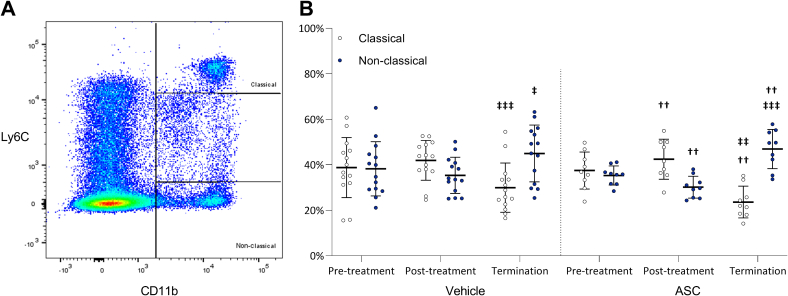
ASC increased the percentage of classical monocytes in hyperglycaemic mice. Peripheral circulating monocyte subsets were assessed in 14 vehicle-treated and 9 ASC-treated mice with flow cytometry (A) on PBMCs collected within 3 days pre- and post-treatment, and at termination (B). The major subsets were Ly6C^hi^ (classical monocytes) and Ly6C^low^ (non-classical monocytes). Differences between the treatment groups were analysed with an unpaired t-test and are represented with *. Differences within the treatment groups were analysed with a paired t-test and are represented with † (when compared to pre-treatment measurements) and ‡ (when compared to post-treatment measurements). Data is represented as mean±SD. Significance was reached with a p-value<0.05*; *p*<0.01**; *p*<0.001***.

To determine the effect of ASC therapy on the distribution of peripheral circulating monocyte subsets, PBMCs were collected within 3 days prior to and 3 days post-treatment and at termination, and analysed for CD11b+/Ly-6C^high^ pro-inflammatory classical and CD11b+/Ly-6C^low^ anti-inflammatory non-classical monocyte subsets through flow cytometry (Fig. 4A).

Prior to treatment in the vehicle- and ASC-treated groups, the percentages of classical (respectively 38.7±13.2 and 37.5±8.1) and non-classical (respectively 38.2±11.9 and 35.3±4.2) monocytes were equivalent (Fig. 4B). Three days post vehicle treatment the percentages classical (41.9±8.7) and non-classical (35.3±8.0) monocytes did not differ significantly compared to pre-treatment. In contrast, in the ASC-treated mice the percentage of classical monocytes was significantly higher (42.4±8.8, *p*=0.005) and the percentage of non-classical monocytes was significantly lower (30.1±4.8, *p*=0.002), compared to pre-treatment.

In vehicle-treated mice at termination, the percentage of classical monocytes (29.9±10.9) was significantly decreased, while the percentage of non-classical monocytes (44.9±12.5) was significantly increased compared to post-treatment only. Similarly, in the ASC-treated group a significant decrease of classical (23.6±7.0) and a significant increase of non-classical (46.9±8.6) monocyte percentages were observed at termination compared to pre-treatment (*p*=0.003 and p=0.002 respectively) and post-treatment (*p*=0.006 and *p*=0.0005 respectively). However, there were no significant differences when directly comparing the ASC groups to the corresponding vehicle groups.

## 4 Discussion

MSC therapy has shown therapeutic potential for atherosclerosis via lowering plaque size and increasing plaque stability in non-DM normoglycaemic animal models [9,13]. We now show that adipose-derived MSCs (ASC) did not affect atherosclerotic plaque size or intra-plaque inflammation in a hyperglycaemic mouse model, when using a clinically relevant MSC dose. Notably, we did observe a borderline significant increase in intra-plaque fibrosis in the ASC-treated mice as well as non-significant trends towards lower intimal CD45+ leucocyte- and adventitial mast cell densities. Lastly, although the percentages of classical and non-classical peripheral circulating monocytes appeared to be more affected by ASC therapy than vehicle treatment within 3 days post-treatment, their levels did not differ significantly at any time point compared to vehicle-treated mice.

Atherosclerotic plaque stability is negatively affected by increased intra-plaque inflammation and subsequent thinning of the fibrous cap, as these processes make the plaque more prone to rupture [5]. Previous studies found that MSC therapy significantly decreased plaque size and macrophage plaque content in non-DM atherosclerotic mice [9,13,[26], [27], [28]]. Additionally, we have previously shown ASC therapy (500,000 ASCs per mouse) improved atherosclerotic plaque stability in non-DM ApoE^−/−^ mice via increasing fibrotic cap thickness and decreasing the percentage of intra-plaque pan-macrophages, without affecting plaque size [25]. Moreover, we observed a higher percentage of peripheral circulating non-classical anti-inflammatory monocytes 4 weeks post-treatment. In the current study in hyperglycaemic ApoE^−/−^ mice (100,000ASCs per mouse), despite non-significant higher levels of intra-plaque fibrosis and lower intimal leucocyte and adventitial mast cell densities, we did not find significant improvement of plaque stability due to ASC therapy. This may have several causes.

Firstly, it is known that hyperglycaemia can affect ASC/MSC function, including their paracrine effect. Both murine and clinical studies found that ASCs isolated from T2DM individuals had a more inflammatory phenotype (e.g., increased *TNF* gene expression) and a reduced immunosuppressive capacity (e.g., decreased *TGFB* expression) [[29], [30], [31], [32]]. However, rather than isolating ASCs from DM mice, we isolated the ASCs from non-DM mice for application in hyperglycaemic mice. Interestingly, Liu *et al.* showed that non-DM ASCs that were exposed to conditioned medium of T2DM ASCs displayed a similar pro-inflammatory phenotype as T2DM-derived ASCs, depicted by increased expression of MHC-II and co-stimulatory molecules CD40 and CD80 [33]. Furthermore, exposure of ASCs derived from non-DM patients to high glucose levels in vitro induced increased cell senescence, decreased cell proliferation and altered cell differentiation [34,35]. Of note, Cramer *et al.* (2010) concluded that impaired stem cell function occurred only in levels of 500 mg/dL and 1000 mg/dL [34], which convert to 27.8 mmol/L and 55.6 mmol/L respectively. In our STZ-induced hyperglycaemic mice, 27.8 mmol/L was almost always reached. The minimal effect of ASC therapy on atherosclerotic plaque characteristics in hyperglycaemic mice in this study compared to previously shown effects in non-DM mice may therefore be partly due to a hyperglycaemia-induced inhibition of therapeutic ASC activity.

Secondly, in our previous study we injected ASC-microbubble complexes (we termed StemBells) rather than ASCs alone [25]. We had shown previously that, compared to ASCs alone, coupling MSCs with ultrasound microbubbles and transthoracic applied ultrasound improved the outcome after experimental myocardial infarction in rats [36]. It is thus possible that the application of StemBells contributed to a greater therapeutic effect on atherosclerosis in our previous study [25]. Previous studies employing systemic application of bone marrow-, skin- or gingiva-derived MSCs alone to affect atherosclerosis found significantly decreased plaque sizes, decreased macrophage plaque content, and a switch towards an anti-inflammatory macrophage (M2) phenotype in atherosclerotic mice [13,[26], [27], [28]]. However, in most of these studies MSCs were provided either prior to atherosclerosis induction [13] or at multiple time points after atherosclerosis induction [9,26,28], whereas in our study ASC treatment was given at one time point after atherosclerosis was established. This may indicate that ASC/ MSC therapy in established atherosclerosis may not necessarily decrease plaque size, but rather decrease plaque instability, and additionally might require multiple time points of treatment for increased effect.

Although these findings suggest clinical benefits of MSC therapy for atherosclerotic patients, it should be noted that murine MSC therapy comprises a higher dose than the MSC therapy applied in clinical trials [[17], [18], [19], [20], [21]]. Our results show that a clinically relevant dose of 100,000 MSCs per mouse (as opposed to the generally applied dose of 0.5–1.5 million MSCs per mouse) did not significantly affect atherosclerotic plaque characteristics in hyperglycaemic ApoE^−/−^ mice. Interestingly, Lin *et al.* did observe a slightly decreased plaque size in the aortic endothelium using as little as 200,000 BMSCs per mouse for MSC treatment [27]. In addition to their dose being twice as high and their different plaque analysis, they found this improvement one week post-injection. It is therefore possible we were unable to detect this temporary improvement as we looked at aortic plaque characteristics four weeks post-treatment. Nevertheless, our findings do corroborate the notion that low effectiveness of MSC therapy in clinical trials compared to pre-clinical studies so far, may be partly due to the relative low cell dose used.

Our study aimed to investigate the effect of ASC therapy on atherosclerotic plaque characteristics in a hyperglycaemic mouse model, using a clinically relevant dose. Despite the borderline significant improvements we observed, we cannot determine from these results if ASC therapy has the potential to improve atherosclerotic plaque stability in a diabetic environment. Further research is therefore warranted to assess the possibilities and limitations of ASC/MSC therapy in DM models, in order to improve ASC/MSC therapy for clinical use.

## CRediT authorship contribution statement

**Amber Korn:** Formal analysis, Investigation, Methodology, Writing – original draft, Writing – review & editing. **Suat Simsek:** Conceptualization, Funding acquisition, Supervision, Writing – review & editing. **Mitchell D. Fiet:** Formal analysis, Writing – review & editing. **Ingeborg S.E. Waas:** Formal analysis, Writing – review & editing. **Hans W.M. Niessen:** Conceptualization, Funding acquisition, Supervision, Writing – review & editing. **Paul A.J. Krijnen:** Conceptualization, Funding acquisition, Supervision, Writing – review & editing.

## Declaration of competing interest

The authors declare that they have no known competing financial interests or personal relationships that could have appeared to influence the work reported in this paper.

## Data Availability

All data relevant to the study are included in the article.
